# Six-Letter DNA Nanotechnology: Incorporation of **Z**-**P** Base Pairs into Self-Assembling 3D Crystals

**DOI:** 10.1021/acs.nanolett.4c03949

**Published:** 2024-10-29

**Authors:** Simon Vecchioni, Yoel P. Ohayon, Carina Hernandez, Shuichi Hoshika, Chengde Mao, Steven A. Benner, Ruojie Sha

**Affiliations:** † Department of Chemistry, New York University, New York, New York 10003, United States; ‡ Foundation for Applied Molecular Evolution, Alachua, Florida 32615, United States; § Department of Chemistry, Purdue University, West Lafayette, Indiana 47907, United States

**Keywords:** DNA nanotechnology, DNA crystallography, unnatural base pairs, X-ray diffraction

## Abstract

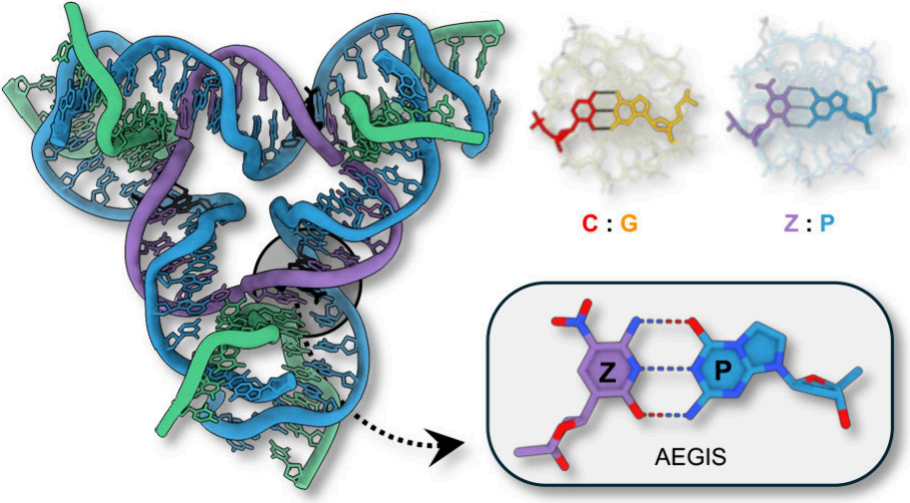

Artificially expanded genetic information systems (AEGIS) were developed to expand the diversity and functionality of biological systems. Recent experiments have shown that these expanded DNA molecular systems are robust platforms for information storage and retrieval as well as useful for basic biotechnologies. In tandem, nucleic acid nanotechnology has seen the use of information-based “semantomorphic” encoding to drive the self-assembly of a vast array of supramolecular devices. To establish the effectiveness of AEGIS toward nanotechnological applications, we investigated the ability of a six-letter alphabet composed of A:T, G:C and synthetic **Z**:**P** (**Z**, 6-amino-3-(1′-β-d-2′-deoxy ribofuranosyl)-5-nitro-(1*H*)-pyridin-2-one; **P**, 2-amino-8-(1′-β-d-2′-deoxyribofuranosyl)-imidazo-[1,2a]-1,3,5-triazin-(8*H*)-4-one) base pairs to engage in 3D self-assembly. We found that crystals could be programmably assembled from AEGIS oligomers. We conclude that unnatural base pairs can be used for the topological self-assembly of crystals. We anticipate the expansion of AEGIS-based nucleic acid nanotechnologies to enable the development of novel nanomaterials, high-fidelity signal cascades, and dynamic nanoscale devices.

The amount of information stored in DNA, with just four nucleotides (GACT), is higher than in a binary code (101110101), but less than it might be if synthetic biologists succeed in adding the independently replicating nucleotides to genetic systems that are possible within the Watson–Crick rules where “small” 6-ring systems pair with “large” 5,6-ring systems with complementary hydrogen bonding patterns. Within standard bonding rules involving hydrogen, nitrogen, oxygen, and carbon atoms, a total of 12 independently replicable atoms are possible, forming six orthogonal nucleobase pairs.

Significant effort has been expended to expand the genetic alphabet.^1^ Expanded amino acid codon systems have been implemented for over 20 years, while lexical expansion of DNA was successfully carried out enzymatically in 1989.^2,3^ Toward the development of synthetic cells and biologically active information carrying molecules, there has been rapid development in reversible, thermodynamically similar nucleobases that do not perturb the overall features of the double helix. Indeed, expanded genetic systems are most likely to work with natural enzymes if the added nucleotide pairs have geometries that are similar to those displayed by standard duplex DNA. Further, polymerases appear to scan the minor groove for electron density.^4^ This density comes from the exocyclic C=O groups of thymine (T) and cytosine (C), and from N3 of adenine (A) and guanine (G). This electron density may also participate in the spine of hydration in the minor groove of the DNA double helix.^5^

Recent work has established an artificially expanded genetic information system, based on eight letters of DNA, a “hachimoji” system.^6^ Two of the four extra hachimoji nucleotides include pyrimidine analogue “**Z**” (6-amino-5-nitro-3-(1′-β-d-2′-deoxyribofuranosyl)-2(1*H*)-pyridone), which forms a stable base pair with purine “**P**” (2-amino-8-(1′-β-d-2′-deoxyribofuranosyl)-imidazo[1,2-*a*]-1,3,5-triazin-4(8H)-one)).^7^ This **Z**:**P** base pair was designed to hydrogen bond under the Watson–Crick rules (Scheme 1). Likewise, both **Z** and **P** present electron density in the minor groove. Accordingly, of all the pairs that might expand the four letter genetic alphabets, these have proven to be the most compatible with existing polymerases.^5^

**Scheme 1:**
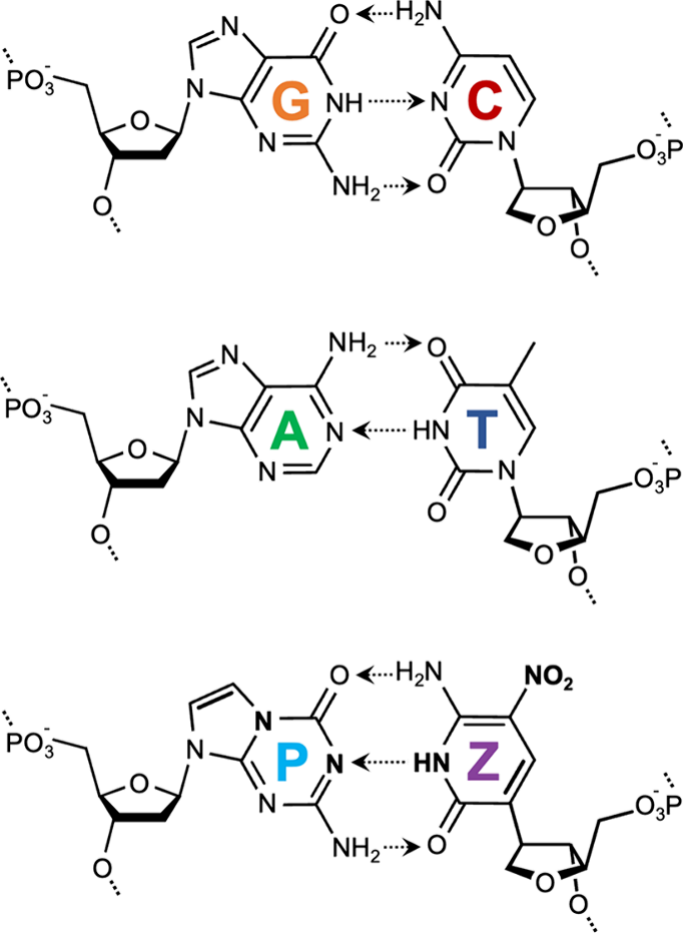
Six-Letter DNA Alphabet Used in This Study G:C and A:T pairs employ canonical Watson–Crick parity, while the **Z**:**P** pair possesses an orthogonal hydrogen bond donor–acceptor combination. Salient **Z**:**P** moieties are shown in bold for emphasis.

In particular, **Z**:**P** base pairs were added to a library of oligonucleotides used for *in vitro* evolution. This six-letter alphabet was used to deliver molecules that bind selectively to liver cancer cells, but not to untransformed liver cells.^8^ Other studies developed AEGIS oligomers that bind selectively to breast cancer cells, capture anthrax proteins, and catalyze chemical reactions.^9−11^ Still others have been incorporated into **Z** and **P** into aptamers conjugated with linear nanotrains that deliver drugs selectively to cancer cells.^12^

To manage its reactivity, a nitro group was appended to the **Z** nucleobase at its C5 position. Further, to obtain the correct hydrogen bonding pattern under the favored tautomer, a carbon replaces the N1 nitrogen, making the nucleobase a “C-glycoside”. The **P** base has shifted a nitrogen from the N7 to N5 position. Together, the pair has a standard “edge on” Watson–Crick geometry but is joined by rearranged hydrogen bond donor and acceptor groups.^5^ The pairing is orthogonal to the pairing of standard bases as a result of the shuffling of its hydrogen bonding units. For example, **Z** presents a donor–donor–acceptor pattern of hydrogen bonding units to a complementary **P** nucleotide, which presents an acceptor–acceptor–donor pattern of hydrogen bonding units (Scheme 1). The crystal structure of a duplex containing **Z**:**P** base pairs was solved and demonstrated overall isomorphism under duplex conditions.^5^**Z**:**P**-containing oligonucleotides have the conformational plasticity of natural DNA, including both A and B forms, a plasticity that may be necessary for their function in living cells.

We were curious to learn whether we could use AEGIS nucleobases for the construction of complex DNA nanomaterials. To carry out this investigation, we employed the tensegrity triangle, a reliable DNA motif which self-assembles into 3D crystals with rationally designed symmetry.^13,14^ This motif has been used to harbor noncanonical components such as triple helical switches,^15^ cyanine dye relays,^16^ and reconfigurable purine-pyrimidine mismatches.^17,18^ Recent work has shown the ability of this DNA motif to elucidate the biomolecular structure of helical modifications including metal-mediated base pairs utilizing modified nucleobases.^19,20^ We previously confirmed a stable metal-coordination base pair between AEGIS nucleotide 5-methyl-isocytosine (**S**) and cytosine that was mediated by Ag^+^ ions in a 3D construct. This study however did not rely upon Watson–Crick parity, but rather leveraged pyrimidine-pyrimidine mispairs to bind linear coordinating transition metal ions, as reported previously.^21^ It remained unclear whether the lexical expansion to six complementary nucleotides, forming three orthogonal base pairs, would disrupt the crystallinity of self-assembled complex matter.

In this paper, the six pairing nucleobases were incorporated into the tensegrity triangle motif in order to show that they behave similarly to the natural nucleic acids, exhibiting the conformational diversity to self-assemble into 3D macromolecular objects. This represents the first case where a nonduplex structure has incorporated any component of any artificially expanded genetic information system by Watson–Crick parity.

In this study, a “two-turn” tensegrity triangle, possessing 21 bp per edge, was designed with monomer C:G sticky ends and 5′-phosphate attached to the helical strands (Figure 1A, green strands). This basic design was modified by adding **Z** and **P** at strategic positions within the triangle core, where stability is highest and crystallographic B-factors have been shown to be minimized.^20,22^ Oligonucleotides incorporating **Z** modifications exhibited green fluorescence and can be visualized in solution, and after crystallization to confirm Z integration (Figure 1B–D).^1^

**Figure 1:**
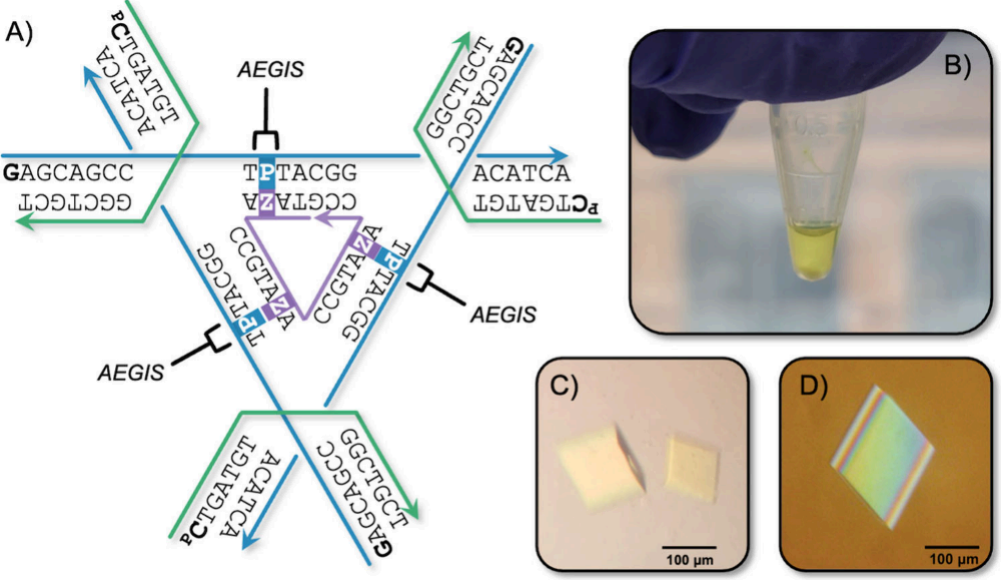
Design and self-assembly of tensegrity triangles containing AEGIS base pairs. (A) One site on each triangle edge was modified to contain a Z:P pair. (B) The **Z** nucleotide possesses a natural green color, which can be observed after self-assembly into 3D crystals both (C) without and (D) with cross-polarization.

Overall, we confirm that the morphology of the crystal is isomorphic to the other two turn tensegrity triangle crystals. We then performed X-ray diffraction on these constructs to confirm the structural integrity of the AEGIS motif. Diffraction was obtained to 3.56 Å, and data were processed into the *R*3 space group, typical of well-constructed tensegrity triangles. A search model based on the unmodified analogue, PDB ID 5W6W, was used to solve the structure by molecular replacement (Figure 2).^23^ The AEGIS tensegrity triangle structure was deposited under PDB ID 8UY5.

**Figure 2:**
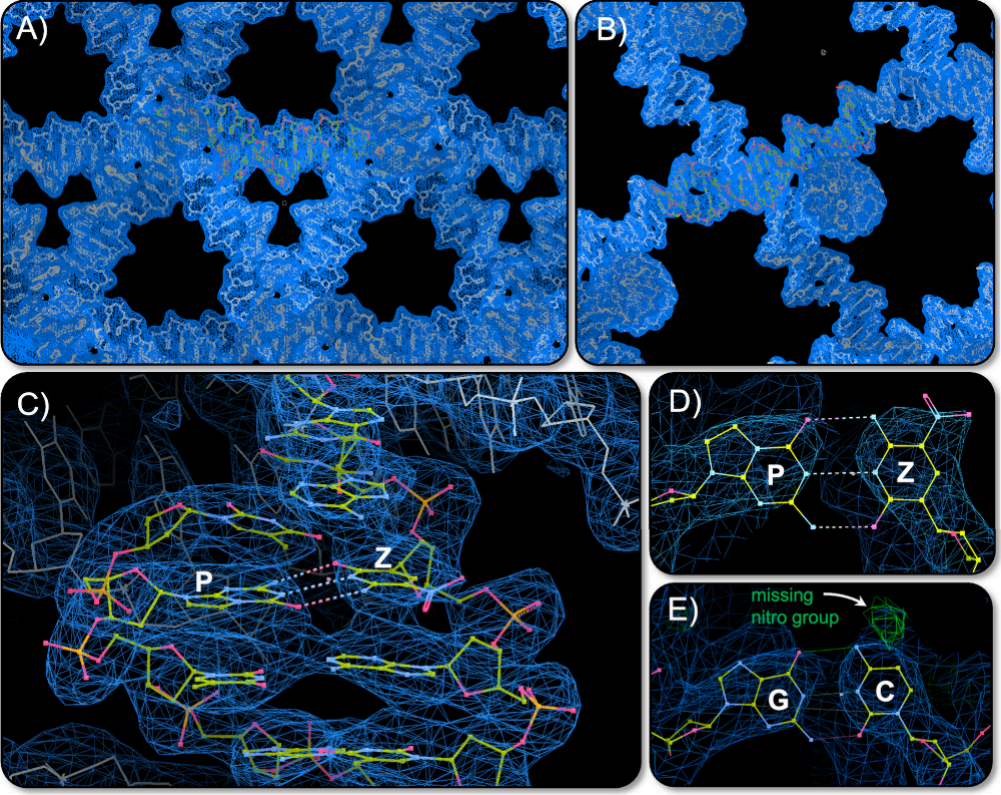
X-ray diffraction of **Z**:**P**-modified tensegrity triangles. (A) Whole unit cell *2F*_o_ – *F*_c_ density shows long-range lattice construction and good alignment of electron density. (B) Side profile of the unit cell shows the rhombohedral cavities. (C) The Z:P base pair and custom restraints are shown. (D) Density for the nitro group in nucleobase Z is observed, (E) while refinement of the same structure with a G:C pair yields *F*_o_ – *F*_c_ density for the missing nitro group (green).

Since the **Z**:**P** pairs fit in the tensegrity triangle, it can be inferred that they can be of use in the self-assembly of 3D DNA arrays and devices. In a previous study, ZP-rich DNA had many of the same properties as CG-rich DNA.^5,6,24^ In both B- and A-duplexes, previous studies determined that the major groove widths for **Z**:**P** pairs are about 1 Å wider than the G:C pairs. To determine whether the use of AEGIS base pairs in complex motifs had significant geometric implications, we compared helicity of the triangle centers in both AEGIS and canonical tensegrity triangles. The periodicity of the motif imposes a helical twist of 10.5 base pairs per turn (bp/T) across the 21 bp motif, but this does not accurately reflect the contribution of local distortions. We have previously found that the junction region—the branched DNA crossover—represents a tension relaxation point that can absorb differences in twist due to metal base pairs,^20^ sequence mismatches,^18^ or over- and underwound duplex segments.^17,25^ To identify these differences, we computed the helicity of the intrajunction region of the triangle, the center of the motif, both with and without the contribution of the junction valve, as described previously.^20^ We found that, excluding the junctions, the helicity of the 6 bp regions were 9.85 and 9.84 bp/T, for the **Z**:**P** and G:C triangles, respectively, while including the junctions yielded helicity values of 10.34 and 10.35 bp/T, respectively (Figure 3A). As the **Z**:**P** pair in this study is adjacent to the J1 junction, we would expect differences in local geometry to be absorbed and computed by the junction valve.

**Figure 3:**
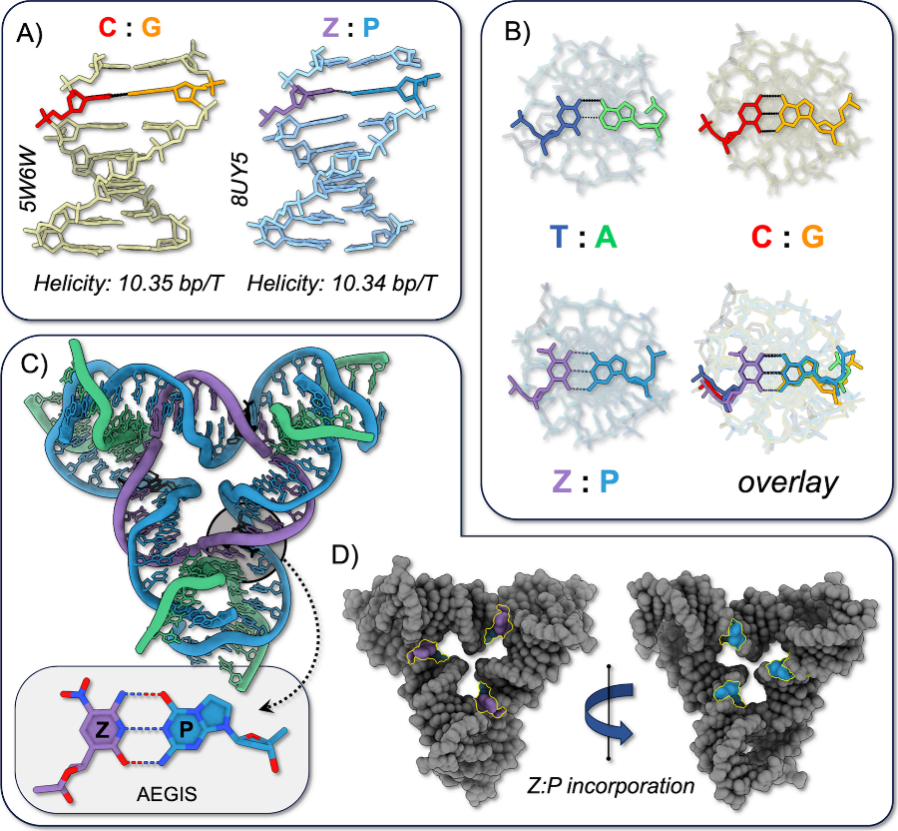
Structural isomorphism between AEGIS and canonical DNA triangles. (A) Helical twists across the inner triangle edge for Watson–Crick and AEGIS triangles are nearly identical, at 10.34 and 10.35 bp/T, respectively. (B) Representative base pairs from the inner triangle are juxtaposed and overlaid to show isomorphism. (C) **Z**:**P** insertion site and structure (inset) are shown and (D) highlighted in the space-filling model from the front and back sides of the triangle.

Structural overlay between the **Z**:**P** and G:C pairs in the two triangles, as well as a distal A:T base pair from the AEGIS triangle, can be seen in the juxtaposition and direct overlay in Figure 3B. We emphasize the nearly isomorphic nature of these base pairs. Figure 3C,D shows the tight integration of the **Z**:**P** pair with the tensegrity triangle, showing good agreement with the rationally designed canonical motif.

The tensegrity triangle serves as a DNA standard for the 3D self-assembly. This motif was modified to include six orthogonally pairing DNA letters, with A:T, G:C, and **Z**:**P** base pairs. The introduction of AEGIS nucleobases was successful, and the overall result was isomorphic to the four-letter Watson–Crick system. To demonstrate the generality of this result, we moved the position of **Z**:**P** base pairs in the DNA tensegrity triangle and found that it had little effect on the formation of DNA motif and crystallization process except placing it on the nick or junction positions. We conclude that the lexical expansion of DNA nanotechnology using well-designed nucleobases can be successfully carried out. Downstream expansion of the DNA alphabet to 8- or even 12-letter systems is anticipated and will rely on the simple but effective validation tools established here. We anticipate that this result will serve as a foundation for a variety of nanostructures, with applications in enzyme obfuscation, data storage, algorithmic assembly, optical circuits, and increasingly complex recognition devices.

## Materials and Methods

### Design, Synthesis, and Purification of DNA

Phosphoramidites of G, C, A, and T were purchased from Glen Research (Sterling, VA). Phosphoramidites of **Z** and **P** were synthesized as reported previously and are available from Firebird Biomolecular Sciences LLC (www.firebirdbio.com).^7^ Oligonucleotides were synthesized by standard phosphoramidite techniques^26,27^ on an Applied Biosystems 394 DNA synthesizer. Sequences can be found in Table S1.

DNA oligonucleotides were purified by denaturing polyacrylamide gel electrophoresis (PAGE) and stained with ethidium bromide. DNA strands were eluted in a solution containing 500 mM ammonium acetate, 10 mM magnesium acetate, and 1 mM EDTA. Eluates were extracted with butanol to remove ethidium, and the DNA was recovered through ethanol precipitation. The DNA was resuspended in water and filtered using a Millipore PVDF 0.22 μm centrifugal filter (Burlington, MA).

### Crystallization and Cryoprotection

Crystals were grown in 5 μL hanging drops containing 0.25 μg/μL DNA, 30 mM sodium cacodylate, 50 mM magnesium acetate, 50 mM ammonium sulfate, 5 mM magnesium chloride, and 25 mM Tris (pH 8.5) equilibrated against a 600 μL reservoir of 1.75 M ammonium sulfate in a thermally controlled incubator. Rhombohedral shaped crystals were obtained with a temperature ramp from 25 to 60 °C at a rate of 5 °C/h, followed by a ramp from 60 to 4 °C at a rate of 0.4 °C/h. Crystals were transferred to a cryosolvent containing 30% glycerol, 100 mM ammonium sulfate, 10 mM magnesium chloride, and 50 mM Tris-HCl (pH 8.5) and subsequently flash-frozen by immersion in liquid nitrogen.

### Data Collection and Processing

A complete sphere of native X-ray diffraction data was collected at Brookhaven National Laboratory using NSLS-II beamline 17-ID-2 (FMX). Data reduction was performed by AutoProc^28^ software (Global Phasing, Cambridge, UK), and anisotropic scaling was performed by a STARANISO^29^ server (Global Phasing) as described in detail previously.^20^ The upper resolution limit was set at local *I*/σ(*I*) ≥ 1.2 along the surface of the diffraction manifold. Diffraction statistics can be found in Table S2.

### Structure Solution

The structure was solved by using a modified 5W6W model as a search probe for molecular replacement using Phaser in the Phenix suite.^30^ Base pair restraints for the **Z** and **P** nucleotides (ligands “DZ” and “DP”) were generated using Matlab (MathWorks, Portola Valley, CA) based on the planarity and parallelity of ideal G:C base pairs. Refinement was supported by ReadySet (Phenix) and carried out using Phenix refinement.

## Data Availability

Structural data were deposited to the Protein Databank under accession code 8UY5. Additional data are available upon reasonable request to ruojie.sha@nyu.edu.

## Supporting Information Available

DNA sequences for the tensegrity triangle, diffraction statistics and custom restraints for DNA crystals (PDF)
