# Supplementary material for: Six-Letter DNA Nanotechnology: Incorporation of Z-P Base Pairs into Self-Assembling 3D Crystals
Source: Nano Lett. 2024 Oct 29;24(45):14302–6. doi: 10.1021/acs.nanolett.4c03949 (PMC11566107; doi:10.1021/acs.nanolett.4c03949)
Supplement: Supplementary file 1 — nl4c03949_si_001.pdf [file nl4c03949_si_001.pdf]

# Supporting Information:

## Six-Letter DNA Nanotechnology: Incorporation of Z-P Base Pairs into Self-Assembling 3D Crystals

Simon Vecchioni[1†], Yoel P. Ohayon[1†], Carina Hernandez[1], Shuichi Hoshika,[2] Chengde Mao,[3] Steven A. Benner[2*], Ruojie Sha[1*]

[1]Department of Chemistry, New York University, New York, NY 10003, USA
[2]Foundation for Applied Molecular Evolution, Alachua, FL 32615, USA.
[3]Department of Chemistry, Purdue University, West Lafayette, IN 47907, USA

†Contributed Equally
*Corresponding authors: R.S. (ruojie.sha@nyu.edu), S.A.B. (sbenner@ffame.org)

**Sequences**

Final motif sequences are shown in **Table S1**. Stoichiometry is indicated for annealing, where a 3:3:1 ratio of strands 1, 2 and 3 is employed, respectively.

| **Table S1:** Oligonucleotide sequences used in this study | | | |
|---|---|---|---|
| *Oligo Name* | *Sequence 5'-3'* | *Type* | *Stoichiometry* |
| ZP-1 | TCTGATGTGGCTGC | Crossover strand (strand 1) | 3 |
| ZP-2 | GAGCAGCCT**P**TACGGACATCA | Helical strand (strand 2) | 3 |
| ZP-3 | A**Z**ACCGTA**Z**ACCGTA**Z**ACCGT | Center strand (strand 3) | 1 |

**P**: 2-amino-8-(1'β−D-2'-deoxyribofuranosyl)-imidazo-[1,2a]-1,3,5-triazin-(8*H*)-4-one
**Z**: 6-amino-3-(1'-β-D-2'-deoxy ribofuranosyl)-5-nitro-(1*H*)-pyridin-2-one.

## Diffraction statistics

**Table S2.** Data collection and refinement statistics

| | AEGIS Triangle |
|---|---|
| **Data collection** | PDB ID: **8UY5** |
| Wavelength (Å) | 0.979237 |
| Space Group | R3 |
| Cell dimensions | |
| *a* = *b* = *c* (Å) | 68.96 |
| $\alpha = \beta = \gamma$ (°) | 100.8 |
| Resolution (Å) | 20.08 – 3.56 |
| | (4.07 – 3.56) |
| Ellipsoidal diffraction limits[a] (Å) | 3.56, 3.56, 4.55 |
| CC(1/2) | 0.999 (0.579) |
| Completeness ellipsoidal (%)[a] | 78.3 (24.7) |
| Redundancy | 11.3 (11.4) |
| | |
| **Refinement** | |
| Resolution (Å) | 20.08 – 3.56 |
| | (20.08 – 3.56) |
| No. reflections | 2893 (2893) |
| $R_{work}$ / $R_{free}$ | 0.152/0.173 |
| RMS bonds (Å) | 0.008 |
| RMS angles (°) | 1.060 |
| Wilson B-factor | 160.1 |
| Total no. of atoms | 861 |
| Average B, all atoms (Å$^2$) | 180.0 |

*Values in parentheses represent the highest resolution shell.

[a]Data scaling performed with ellipsoidal cutoff using the STARANISO server (Global Phasing), with severe anisotropy observed along the three-fold axis.[1]

**Custom Restraints**

Matlab generated .eff file for for Phenix refine, where residue /A:10 is d**P** and /B:6 is d**Z**:

```
geometry_restraints.edits {
  angle {
    atom_selection_1 = chain A and resid 10 and name C2
    atom_selection_2 = chain A and resid 10 and name N1
    atom_selection_3 = chain B and resid 6 and name N3
    angle_ideal = 116.2
    sigma = 3.46
  }
  angle {
    atom_selection_1 = chain A and resid 10 and name C2
    atom_selection_2 = chain A and resid 10 and name N2
    atom_selection_3 = chain B and resid 6 and name O2
    angle_ideal = 122.2
    sigma = 2.88
  }
  angle {
    atom_selection_1 = chain B and resid 6 and name C4
    atom_selection_2 = chain B and resid 6 and name N4
    atom_selection_3 = chain A and resid 10 and name O6
    angle_ideal = 117.3
    sigma = 2.86
  }
  angle {
    atom_selection_1 = chain B and resid 6 and name C2
    atom_selection_2 = chain B and resid 6 and name O2
    atom_selection_3 = chain A and resid 10 and name N2
    angle_ideal = 120.7
    sigma = 2.20
  }
  angle {
    atom_selection_1 = chain B and resid 6 and name C2
    atom_selection_2 = chain B and resid 6 and name N3
    atom_selection_3 = chain A and resid 10 and name N1
    angle_ideal = 115.8
    sigma = 2.88
  }
  angle {
    atom_selection_1 = chain A and resid 10 and name C6
    atom_selection_2 = chain A and resid 10 and name O6
    atom_selection_3 = chain B and resid 6 and name N4
    angle_ideal = 122.8
    sigma = 3.00
  }
  bond {
    action=add
    atom_selection_1 = chain A and resid 10 and name N2
```

```
      atom_selection_2 = chain B and resid 6 and name O2
      distance_ideal = 2.8
      sigma = 0.2
    }
    bond {
      action=add
      atom_selection_1 = chain A and resid 10 and name N1
      atom_selection_2 = chain B and resid 6 and name N3
      distance_ideal = 2.8
      sigma = 0.2
    }
    bond {
      action=add
      atom_selection_1 = chain A and resid 10 and name O6
      atom_selection_2 = chain B and resid 6 and name N4
      distance_ideal = 2.8
      sigma = 0.2
    }
    parallelity {
      action = add
      atom_selection_1 = chain A and resid 10 and (name C2 or name N2 or name C6 or name O6 or name
N1 or name N5 or name C4 or name N3 or name N9 or name C8 or name C7)
      atom_selection_2 = chain B and resid 6 and (name C2 or name O2 or name C1 or name N3 or name
C4 or name N4 or name C5 or name N)
      sigma = 0.027
      target_angle_deg = 0
    }
    planarity {
      action = add
      atom_selection = (chain A and resid 10 and (name C2 or name N2 or name C6 or name O6 or name
N1 or name N5 or name C4 or name N3 or name N9 or name C8 or name C7)) or \
                       (chain B and resid 6 and (name C2 or name O2 or name C1 or name N3 or name C4 or
name N3 or name C5 or name N))
      sigma = 0.176
    }
  }
}
```

References

1. Tickle, I.; Flensburg, C.; Keller, P.; Paciorek, W.; Sharff, A.; Vonrhein, C.; Bricogne, G., Staraniso. *Cambridge, United Kingdom: Global Phasing Ltd* **2018**.
